# Community-academic partnerships in HIV-related research: a systematic literature review of theory and practice

**DOI:** 10.7448/IAS.18.1.19354

**Published:** 2015-01-27

**Authors:** Ulrike Brizay, Lina Golob, Jason Globerman, David Gogolishvili, Mara Bird, Britt Rios-Ellis, Sean B Rourke, Shirin Heidari

**Affiliations:** 1 International, AIDS Society, Geneva, Switzerland; 2 Ontario HIV Treatment Network, Toronto, Canada; 3 Center for Latino Community Health, Evaluation and Leadership Training and California State University Long Beach Long Beach, CA, USA; 4 Ontario HIV Treatment Network, St. Michael's Hospital and University of Toronto, Toronto, Canada; 5 Inforia, Geneva, Switzerland

**Keywords:** HIV, action research, participatory action research, community-based research, community-based participatory research, community involvement, literature review

## Abstract

**Introduction:**

Community involvement in HIV research has increased over recent years, enhancing community-academic partnerships. Several terms have been used to describe community participation in research. Clarification is needed to determine whether these terms are synonymous or actually describe different research processes. In addition, it remains unclear if the role that communities play in the actual research process follows the recommendations given in theoretical frameworks of community-academia research.

**Objectives:**

The objective of this study is to review the existing terms and definitions regarding community-academic partnerships and assess how studies are implementing these in relation to conceptual definitions.

**Methods:**

A systematic literature review was conducted in PubMed. Two reviewers independently assessed each article, applying the following inclusion criteria: the article must be published in English before 2013; it must provide an explicit definition and/or defining methodology for a term describing research with a community component; and it has to refer to HIV or AIDS, reproductive health and/or STDs. When disagreements about the relevance of an article emerged, a third reviewer was involved until concordance was reached. Data were extracted by one reviewer and independently verified by a second. Qualitative data were analyzed using MaxQDA for content and thematic analyses while quantitative data were analyzed using descriptive statistics. Community feedback on data analysis and presentation of results was also incorporated.

**Results:**

In total, 246 articles were retrieved, 159 of which fulfilled the inclusion criteria. The number of studies that included community participation in the field of HIV research increased between 1991 and 2012, and the terms used to describe these activities have changed, moving away from action research (AR) to participatory action research (PAR), community-based research (CBR) and community-based participatory research (CBPR), with the latter being the most commonly used term. While definitions of all terms had common characteristics (e.g. participation of community in research process), they varied with regard to the emphasis placed on these characteristics. The nature of community participation in reviewed studies differed considerably from that described in theoretical models.

**Conclusions:**

This study indicates the increase of participatory approaches in HIV research and underlines the need for clarification of terms and a framework providing orientation to community-academia partnerships.

## Introduction

Communities have always played critical roles in responding to the HIV epidemic. Over the past three decades, community-based organizations have been key providers of HIV prevention, treatment, care and support services [1]. Community stakeholders have also been crucial in addressing the social, political, legal and financial environment needed to support the scale-up of effective responses [2]. During this time, community-academic partnerships have gained increasing prominence in HIV research, but a commonly accepted definition and a set of criteria for research with a community component are currently unavailable.

Even though key opinion leaders in the HIV field advocate for increased participation of communities in research activities and refer to community involvement in their publications [3–6], there is no commonly agreed definition for research with a community component. Moreover, several terms are used to label such collaborative research (The term collaborative research will be used in this article exclusively to describe research involving partners from community and academia). These terms include for example, community-based research (CBR), community participatory research (CPR), community-based participatory research (CBPR), action research (AR), and participatory action research (PAR). Clarification is needed on whether each term is synonymous or whether they actually describe different research processes.

In the general literature on CBR, the work of Israel *et al*. has provided a critical template for the core dimensions of CBR [7]. Together, these dimensions have given shape to a “working definition” of CBR:

CBR in public health is a collaborative approach to research that equitably involves all partners (for example, community members, organizational representatives, and researchers) in all aspects of the research process. The partners contribute unique strengths and shared responsibilities to enhance understanding of a given phenomenon and the social and cultural dynamics of the community, and integrate the knowledge gained with action to improve the health and well-being of community members. [7]

This definition provided by Israel *et al*. is only one of many definitions for research involving community cited in the literature. Although many other definitions include similar components, the diversity of terms, definitions and key characteristics indicate a lack of consistency. In addition, there is a need to understand how theoretical concepts of community participation are reflected in the implementation of studies. The lack of clarity in terms and definitions and the call for increased community involvement in research may lead to inaccurate labelling of research as community partnered.

The objective of this literature review was to analyze the theoretical terms and definitions used to describe research involving community and to determine how these conceptual definitions are implemented in HIV research practices.

## Methods

### Literature search

The literature review complies with the guidelines outlined in the PRISMA Statement [8]. A systematic literature search was conducted in PubMed using the search terms listed in Table 1. Only articles published before the search date (December 2012) were included.

**Table 1:** Keywords for PubMed search

“community-based participatory research” [Title/Abstract] OR “community-based research” [Title/Abstract] OR “community participatory research” [Title/Abstract] OR “participatory action research” [Title/Abstract] OR “community collaborative research” [Title/Abstract] OR “community partnered research” [Title/Abstract] OR “CBR” [Title/Abstract] OR “CBPR” [Title/Abstract] OR “community engaged research” [Title/Abstract] OR “action research” [Title/Abstract] OR “community empowerment research” [Title/Abstract]) AND (“HIV” [All Fields] OR “AIDS” [All Fields] OR “reproductive health” [All Fields] OR “sexually transmitted” [All Fields] OR “STD” [All Fields]) NOT “action research arm test” [Title/Abstract]

In addition, websites of research institutes, and community or international organizations were reviewed to capture existing definitions. These included the Community Based Research Centre; the Elizabeth Glaser Paediatric AIDS Foundation; the Global Fund to Fight AIDS, Tuberculosis and Malaria; the Ontario HIV Treatment Network; the National Institutes of Health Office of AIDS Research; the Social Research Centre in HIV Prevention (University of Toronto); Joint United Nations Programme on HIV/AIDS; United Nations International Children's Emergency Fund; the Wellesley Institute; and the World Health Organization.

### Article selection

The following inclusion criteria were applied: The article must be published in English and provide an explicit definition or a clear description of the research process which illustrates the specific understanding of community-academic research, or both, for a term paraphrased by “research with a community component” and must refer to HIV, AIDS, reproductive health and/or sexually transmitted diseases.

Two reviewers independently assessed each paper for inclusion or exclusion. All documents not written in English and/or without mention of one of the search terms in the abstract or title were excluded during the abstract screening process. When abstracts were unavailable, full text articles were retrieved.

In case of disagreement regarding the relevance of an article, a third independent reviewer arbitrated the decision. All full text articles were retrieved and assessed.

### Data extraction

Data were extracted into a standardized Excel spreadsheet by one reviewer and independently verified by a second. Data were collected in the categories detailed in Table 2.

**Table 2:** Literature review data extraction

1. Article: Author, title 2. Year of publication 3. Publication type 4. Term used (PAR, AR, CBR, CBPR or other) 5. Original definition of term 6. Cited definition of term, including source 7. Period of data collection 8. Country (where study took place) 9. Objective of article/study 10. Study type 11. Study methodology 12. Study target group 13. Who was the community partner?	14. Community advisory board 15. Role of community partner 16. Who took initiative for the research? 17. Results dissemination to community 18. Institutional review board (IRB) approval 19. Which institution gave IRB approval? 20. Ethical approval by community 21. Which capacity-building activities took place? 22. What was the added value of the community involvement? 23. What were the negative aspects/limitations of community involvement? 24. Did the community involvement lead to changes in the community? 25. Funding agency 26. Other partners involved

### Data analysis

Qualitative data were analyzed using a grounded-theory approach [9]. Codes were developed based on a representative sample of articles, which were subsequently applied to the whole dataset using qualitative data analysis software (MaxQDA). Quantitative data were analyzed using descriptive statistics. Feedback from a community representative on data analysis and presentation of results was also incorporated. (Table 3)

**Table 3:** Methods and objectives for data analysis

Quantitative data	Qualitative data
Term development: Term used in reference to the article's year of publication (1991–2012). *To display the use of the various terms over time*. Study type associated with term: Term used in reference to the study type (qualitative, quantitative, mixed methods). *To identify potential patterns in practical usage*. Institutionalization of community component: *To explore if existence of a community advisory board is a significant feature of research with a community component*. Initiation of research: Academia- or community-initiated endeavour or both equally involved. *To identify a potential pattern of research projects with community component*. Dissemination of research results to the community: Dissemination among academia, among community or both. *To identify the main audience of findings*. Ethical approval by the community: *To identify if there are formal mechanisms to take the community's ethical requirements for research into consideration for the study*.	Term definition: Definition or defining methodology of term as provided in the article. *To identify existing terms and definitions, similarities and differences and patterns of practical usage*. Study methodology (also in reference to term used): Methodology applied during study. *To identify most frequently used methods in practical use of research with a community component*. Role of community: The depth and width of the community's role in research with community component. *To compare the role allocated to the community in the definition of the term with the research practice*. Capacity building: *To identify whose research capacity (community, researcher or both) is built, what types of capacity building take place and if there is an initial intention of capacity building in research studies with a community component*. Added value of community component: *To identify if there is added value of a community component and, if yes, of what value it is*. Negative aspects/limitations of community component: *To identify challenges specific to research with a community component*.

## Results

**Figure 1:**
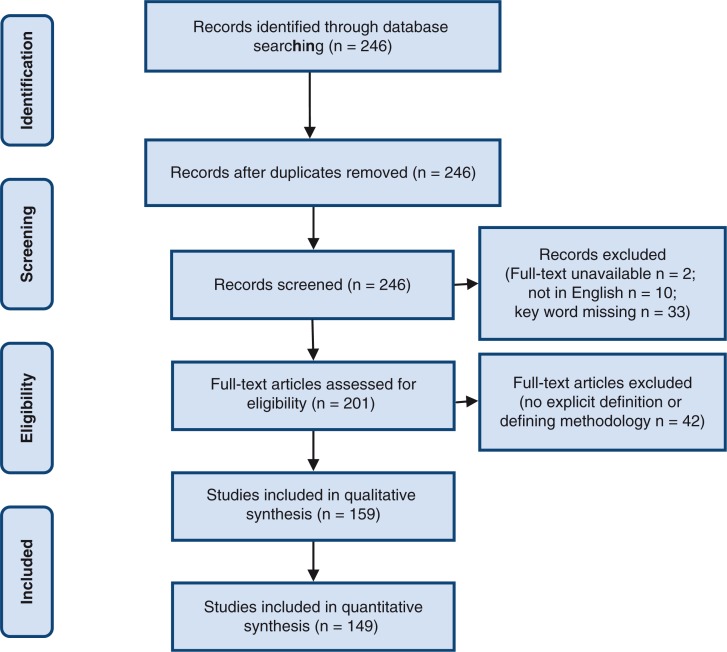
PRISMA 2009 flow diagram.

The key word search resulted in 246 articles. Each article was assessed independently by two reviewers for eligibility; there was 93% concordance between reviewers. Thirty-four percent of the articles found were excluded (87 of 246). All included articles (159) were analyzed qualitatively, but only original research articles (149) were included in the quantitative analysis. Detailed information regarding reasons for exclusion is available in Figure 1. A list of all included articles can be viewed in the Supplementary table1.

### Terms and their definitions

Each term used to describe research with a community component was extracted from original research articles. In order to categorize articles, the main term most commonly utilized in each article was identified and constituted the basis for analysis.

The number of studies that included a community component in the field of HIV research has increased from the beginning of the epidemic until 2012. The first article identified by the review was published in 1991. Since the mid-2000s, there has been a surge of published studies in which communities play a crucial role. From 2006 to 2012, an average of 15 articles labelled CBR, CBPR, AR or PAR were published annually with a peak of 25 articles in 2010.

**Figure 2:**
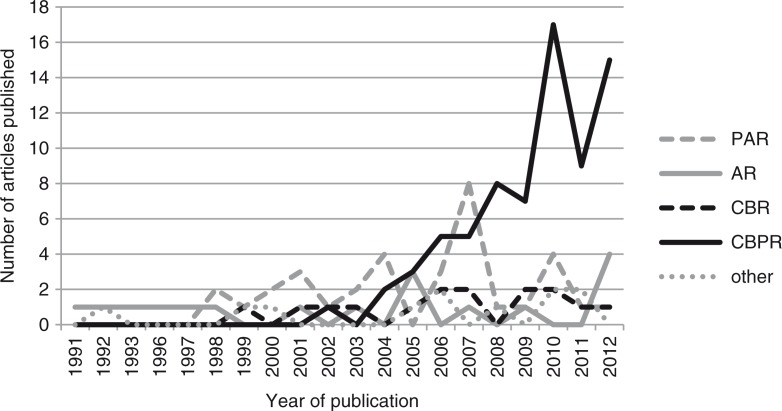
Use of terms between 1991 and 2012.

The terms used to describe studies with a community component have also changed over time (Figure 2). While AR was the predominant term in the 1990s, it was replaced by PAR, CBR and CBPR, with the latter being the most common term at the end of 2012. Several other terms, for example, community-engaged research, collaborative action research and community-partnered participatory research, have been used by individual authors.

While identifying the main term used in each study to describe the research approach, it became apparent that some terms are used synonymously. In the context of HIV research, PAR and AR, as well as CBPR and CBR, are defined in similar ways.

#### Participatory action research and action research

AR strives to actively involve community members in the research process and is the oldest research approach identified in the literature. It was first outlined by Lewin in 1946 when he described the circular process of “planning, action, and fact-finding about the result of the action” [10] that would later become the basis of the AR concept. For Lewin, AR was the necessary evolution of social research as “research that produces nothing but books will not suffice” [10]. The iterating cycle of planning, action and evaluation, as well as social change as an inherent part of the research process, has remained the main characteristic of AR.

While only four publications analyzed in the literature review refer to Lewin [11–14], many based the development of their studies on Stringer, who revitalized AR in the 1990s. Like Lewin, Stringer describes AR as a cyclical process involving different repetitive phases that he named “look, think, act” [15]. The first phase refers to the gathering of data to better understand the situation, the second phase refers to the analysis and interpretation of the information collected in the first phase, and the third phase refers to the planning and implementation of activities. Emphasis is placed on the fact that the process is not linear, but circular, which requires a new cycle of the three phases to evaluate the impact of the activities and to modify interventions [15].

An important aspect of AR is the implementation of research findings into programmatic action that leads to social change [16,17]. The iterating process of the research approach enables constant reflection of the intervention and allows for flexibility. This permits the adaptation of planned interventions to a changing environment or varying needs of the target group [18–20]. The applicability of AR for planning, monitoring and evaluation of programmes makes this approach especially valuable for service providers [20].

In addition, the participation of professionals or members of the target group in the research process itself can facilitate a process of reflection that enables participants to question their actions and initiate social change.

PAR uses the same principles as AR, but incorporates a stronger participatory component [21]. While AR is generally planned and implemented by academic researchers, PAR encourages members of the target group to take part in the design and conduct of the study [13,22]. In PAR, “the community is involved in identifying the research questions, developing the project design (data collection and analysis) and in the dissemination of findings” [23].

Similar to AR, the final objective of PAR is the initiation of social change within a community. The participation in the research process and the alternate steps of reflection and action creates a deeper understanding of the situation and raises the consciousness of participants. The heightened awareness lays the basis for social change [24–28].

Beside Lewin and Stringer, one of the roots of PAR is the work of Freire. In his book, *Pedagogy of the Oppressed*, first published in Portuguese in 1968, he describes a research process that contains all the characteristics of PAR without providing a specific name for this research approach. Corresponding with Freire's understanding of teaching, research should help participants gain a deeper understanding of their situation. While Freire does not mention empowerment himself, it can be seen as the feature of PAR that was mostly influenced by his writings. A “deepened consciousness” [29] that has been developed through research can help participants to better understand their situation and to take social action [29]. The notion of empowerment through the participation in PAR is highlighted as an important characteristic of PAR in publications analyzed in the literature review [23,28,30–32].

#### Community-based participatory research and community-based research

While the differences of AR and PAR can still be identified in their theoretical framework, the definitions of community-based research (CBR) and community-based participatory research (CBPR) are less distinctive. The terms CBR and CBPR are employed interchangeably. Important references of CBR and CBPR that authors use to develop their research provide similar definitions for both approaches [7,33]. Likewise, definitions of CBR and CBPR given by authors of articles identified through the literature review highlight the same aspects. Radda *et al*. define CBR as “a process that brings researchers and community members together to collaboratively conduct research on a problem of concern to the community” [34]. Meanwhile, Rhodes *et al*. explain that “CBPR is an approach to research that ensures full and equal participation by community members […] in all aspects of the research process” [35].

The differences in the use of CBR and CBPR are based less on different characteristics and more on changes over time. While CBR has been employed continuously since the end of 1990s in the field of HIV, CBPR has become prevalent since 2005. Even authors who have strongly influenced the definition and use of CBR transitioned in past years to the term CBPR. Israel *et al*. provided a definition of CBR that serves as a template for many participatory studies in the HIV field [7]. However, recent articles by her and her team use the term and provide definitions for CBPR [36–38].

While the aspect of social change and the implementation of findings still play an important role in CBR and CBPR, the creation of knowledge and the scientific rigour of the study are equally important [39–42]. The advantage of CBR and CBPR is based on the collaboration between practice and science for the benefit of academic researchers and community members alike [42].

### Similarities and differences in participatory research approaches

While common definitions of CBPR, CBR, PAR and AR include similar traits, they vary in the significance they place on these characteristics.

Participatory research processes can be divided into three phases: the planning phase, the research phase and the action phase. All definitions highlight the participation of communities in each of these different phases, but the relevance of the involvement in different activities varies depending on the research approach. The review of papers describing participatory research in the HIV field shows that CBPR places the strongest emphasis on the general involvement of community members in all phases of research. In CBR and PAR, this is also highlighted in definitions provided by authors, but plays a lesser role. AR is often designed and implemented by academic researchers, while the community is often restricted to the role of research participants who cannot influence the research procedure and design.

Community involvement in the planning of HIV research plays an equally important role in PAR, CBR and CBPR. Definitions of these approaches describe the community contribution to the identification of the research question and to the design of the research. The involvement, which can take different forms, aims to ensure the relevance of the research from the community's perspective, the suitability of the selected research methods and the acceptability of research instruments. In addition to the identification of the research question and the research design, definitions of CBPR also mention community support in securing funding and ethical approval for the planned study. Planning is the phase that has the least community participation in AR, which confirms that the development of an AR project lies mainly in the hands of academic researchers.

All definitions of participatory research approaches in the HIV field describe community engagement in the research phase. AR and PAR place more importance on the analysis and verification of data by community members and less importance on the conduct of the research. Both aspects are equally significant in definitions of CBR and CBPR. In addition, definitions of CBR and CBPR mention a third aspect which is the facilitation of recruitment of research participants.

Community involvement in AR is especially strong in the action phase. PAR, CBR and CBPR also encourage community participation in the action phase, but definitions reflect more diversity in the specific activities. The community not only is involved in and benefits from activities that are developed based on the research, but also supports dissemination of research results, as well as planning and implementation of interventions. In addition to outlining the role of the community in the research process, definitions highlight some other features that participatory research approaches in the HIV field have in common.

**Figure 3:**
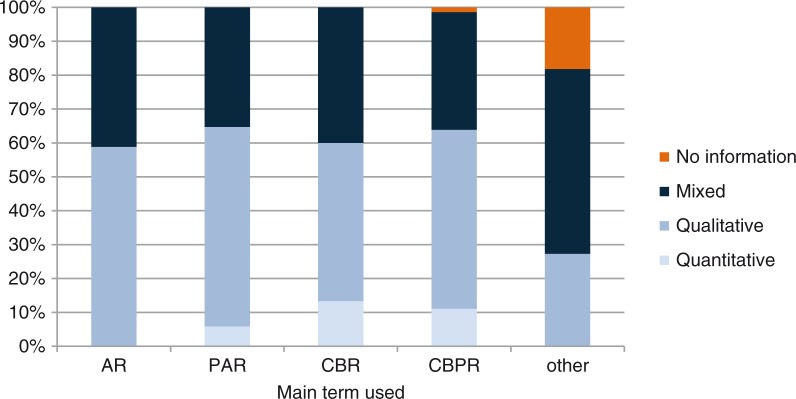
Use of study methods according to term.

CBPR, CBR, PAR and AR are unspecific research approaches in so far as they can be utilized as a framework for diverse studies. They do not stimulate the use of specific methods or research instruments, even though some definitions highlight data collection tools like focus groups or in-depth interviews that are considered especially suitable for collaborative research with community. The majority of studies analyzed in the literature review use qualitative or mixed methods, but the approaches are equally applicable to quantitative studies if the community determines that quantitative methods are the most adapted to answer the identified research question (Figure 3).

All approaches see representatives of community organizations or community members as the source of specific knowledge and insight that can increase relevance of research and validity of results. The role of academic researchers lies in guiding communities in the research process, building research capacity and ensuring scientific rigour of the study. Due to the involvement in all phases of the research process, as described in PAR, CBR and CBPR, community partners are more than research participants. They become community researchers who plan and implement a study with academic researchers in a collaborative manner. Ultimately, collaborative research has the potential to increase the quality and significance of research for the benefit of all parties involved.

### Comparing theory and practice

Despite individual differences in definitions of CBPR, CBR, PAR and AR, all approaches include similar characteristics and show strong correlations in defining the role of the community in the research process. Conversely, when comparing community participation as highlighted in theoretical frameworks of collaborative research with the role they actually played in the analyzed studies, significant differences become apparent.

**Figure 4:**
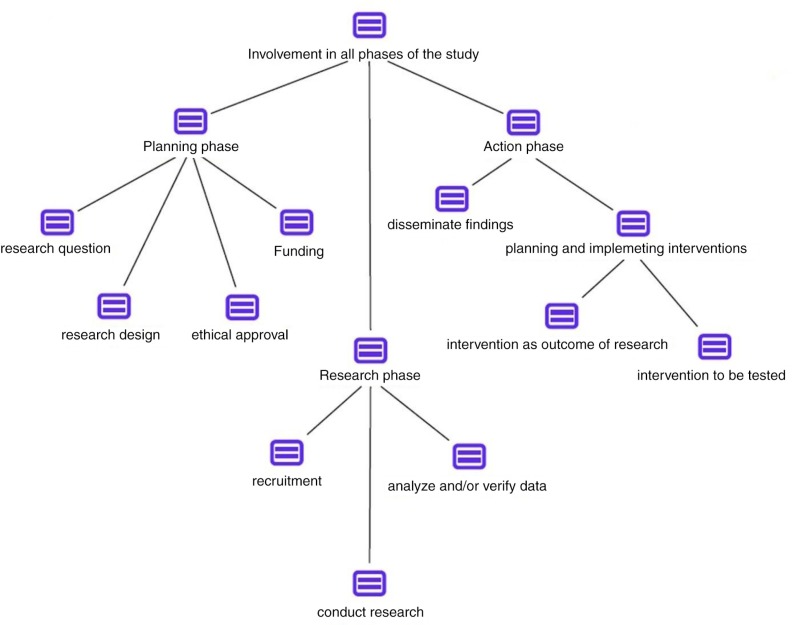
Code tree.

Following codes were developed and used to analyze community involvement in theoretical frameworks and research practices of collaborative HIV research (Figure 4).

**Figure 5:**
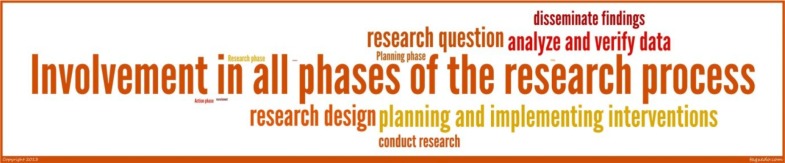
Word cloud theory.

Generally, definitions highlight the involvement of community in all phases of the research process. Other important aspects include identification of the research question, design of the study, analysis and verification of data, and planning and implementation of interventions. Community support of other activities, for example, conduct of research, dissemination of findings, recruitment process, or application for funding and ethical approval, are less often mentioned in definitions. The word cloud in Figure 5 reflects the role of community as described in theoretical frameworks of collaborative research. The size of the different aspects represents the emphasis that is placed on the corresponding activity as coded and analyzed during the literature review.

**Figure 6:**
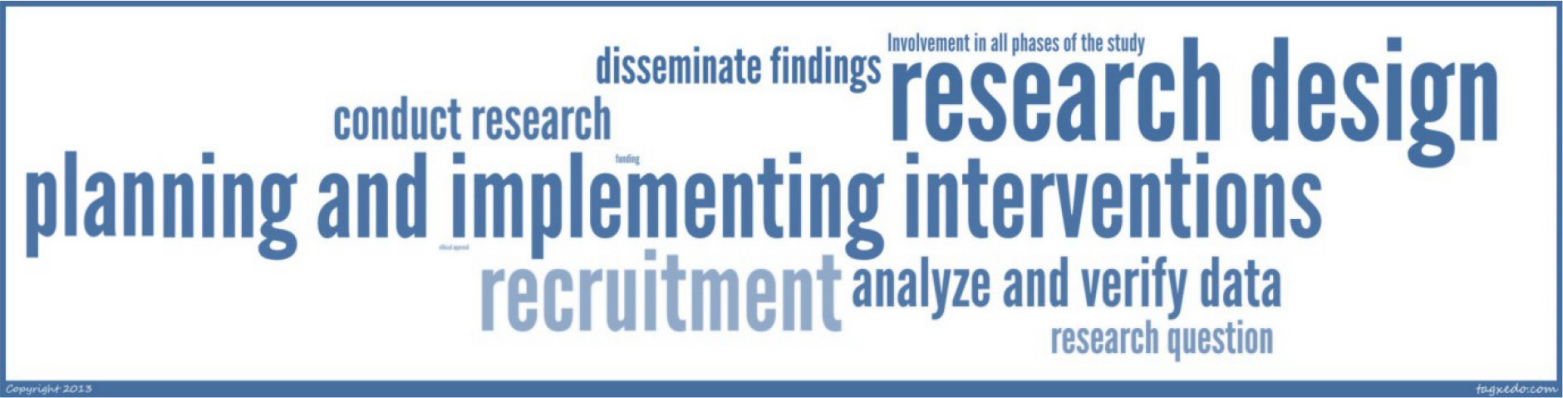
Word cloud: practice.

Using the same codes, analyzing the implementation of the studies and the description of the community role during the actual research process, the results only partially reflect the theory. Interestingly, only a minority of the assessed studies in the HIV field provide an indication that the community was involved in all phases of the research process, even though this aspect can be seen as the fundamental principle of all collaborative research (Figure 6).

In practice, community involvement in HIV research focuses especially on the design of the research, the recruitment of research participants and the planning and implementation of interventions. There are various ways that a community contributes to the planning of the study design. Community members and academic researchers jointly select and/or develop or adapt the data collection instruments, they draft the content of surveys or interviews, or they help pre-test and refine the tools. Based on their insight and knowledge, community members are well positioned to identify research procedures and content that are acceptable to community members and that meet their specific requirements. Similarly, recruitment benefits from the advantageous position and social trust that community representatives and community organizations have within the community. They are often more knowledgeable than academic researchers regarding how and where potential research participants can be reached, which might vary depending on the target population. For example, young men who have sex with men will be able to provide information about virtual chat rooms that are frequented by their peers, and local service providers are well placed to establish contacts with their clients and reduce barriers by providing space to conduct focus group discussions or interviews. The planning and implementation of interventions often lies in the hands of the community. Community organizations play a crucial role in translating research findings into tangible programmes and advocacy activities that benefit community members. Traditionally, the task of academic researchers ends with the distribution of the research findings. Therefore, the initiative of the community is needed to drive the implementation of results to improve existing programmes or provide new services and/or to advocate for policy changes to enable research findings to reach their full potential.

Collaborative studies in the field of HIV involve community members as community researchers stronger than emphasized in theoretical frameworks. The possibilities for the involvement of community researchers are as manifold as the benefits of this practice. Community members moderate group discussions, administer questionnaires and assist in social mapping exercises. The rapport and trust that can be created by involving community members in the conduct of the study enables the generation of more personal data and facilitates the correction of prejudice towards research in general. However, besides these advantages of involving community members in the conduct of the study, several disadvantages were mentioned. While talking to a peer helps overcome barriers to discuss sensitive issues like sexual behaviour or HIV status of participants, it can also create social pressure, prohibit discussion of stigmatized behaviour and raise concern regarding confidentiality. Depending on the situation and target group, the involvement of community members in the generation of data must be carefully planned.

Other aspects of community participation in the implementation of studies, for example, verifying and analyzing data and dissemination of results, correspond to the theoretical model. Again, community members’ specific knowledge and insight enables community stakeholders to validate and interpret findings, which helps increase the quality and accuracy of results. The dissemination of findings beyond the scientific community plays an important role in collaborative research. While traditional research produces knowledge that is only accessible to a limited number of experts, collaborative approaches strive to report their findings back to the community and facilitate their practical use at the community level. Community members are well positioned to communicate the results in a form that is accessible and understandable to their peers, as well as utilize results for political advocacy. Despite this improvement, the distribution of responsibilities in the dissemination of results still reflects the traditional position of academic and community partners. Generally, academic researchers focus on scientific publications, while community stakeholders report back to their communities and use the data for lobbying. Community partners who appear as co-authors of scientific publications still remain the exception.

Community involvement in securing funding and ethical approval seems to play a minor role in theoretical models, as well as in practical implementation. This reflects the structural constraints experienced by many community representatives engaged in HIV research. In order to qualify for a research grant or to submit a request for ethical approval, applicants generally have to be affiliated with an academic institution or need to have certain academic qualifications. Consequently, a power imbalance is created between academic and community partners. Carrying the responsibility to secure funding and to comply with related requirements, the academic institution also maintains authority of the spending of the funds. Similarly, the ethical approval procedure leaves limited space for flexibility during the research process and obliges academic researchers to enforce initial plans despite changing needs within the community.

## Discussion

### Collaborative approaches in HIV research

The literature review demonstrates the evolution in community engagement in HIV research over the past decades. Diverse terms and definitions have been developed to describe research involving community. The review of different theoretical frameworks provides insight into the historic development and conceptual roots of different collaborative approaches. Despite the fact that there are no specific definitions for the context of HIV, authors confirm that participatory research is especially suitable for HIV research. HIV is a major public health concern in many settings and among certain populations. The HIV epidemic reveals the strong social component of the disease, particularly affecting marginalized communities that are often hard to reach through conventional research [43–45].

The many advantages of community involvement in research identified through the literature review can be applied to communities affected by HIV. Involving community improves the quality and relevance of research due to the insider knowledge provided by community members as they help identifying relevant research questions [46,47], developing suitable research instruments [23,48,49] and dissemination tools [21,50–52], and verifying and interpreting collected data [53–55]. Thanks to these approaches in the HIV field, the perception that communities often only serve as objects in conventional research without the possibility of influencing the research process is increasingly challenged. Inclusive approaches can create trust between community and academic partners, and thereby facilitate the research process. More importantly, such inclusive approaches have the potential to lead to the empowerment of people living with and affected by HIV, and result in tangible intervention to address the needs of the community.

Despite the many advantages in involving community in HIV research, several challenges have also been reported. Including community as an equal partner in the research process can be resource-intensive as resources are required to build the partnership and achieve consensus regarding the research process. It takes time to build the research capacity of community partners as well as the contextual understanding of community realities by academic researchers who may be attached to research protocols and unable to respond to community needs. The literature review raised several issues that merit further investigation. These include, but are not limited to, gender differentials in community participation, ethical considerations, barriers to equal community involvement and advantages of community-partnered research.

### Diversity of terms and definitions

**Figure 7:**
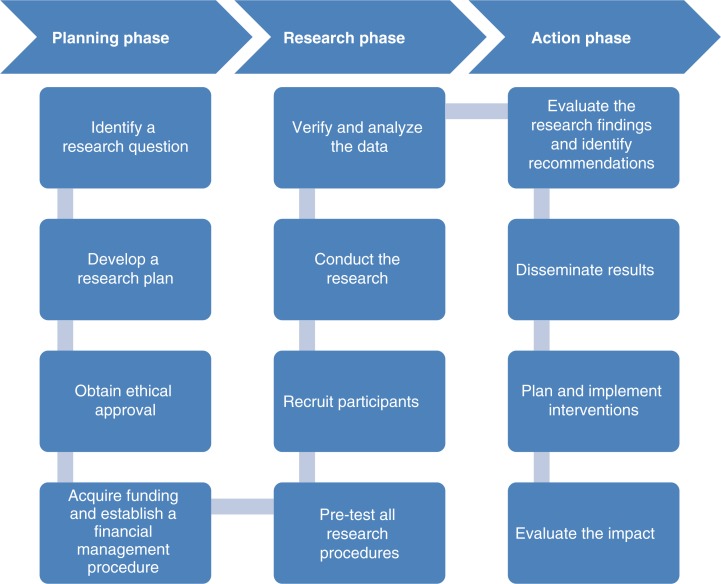
Collaborative research.

While the emergence of a multitude of terms and definitions describing HIV research involving community reflects the importance it has gained in recent years in the field, the diversity of definitions create a complexity that hampers a structured implementation in line with established conceptual theories. The plurality of theoretic frameworks leaves room for different interpretations of the role of communities in research. In the literature review, not all research self-labelled as community-partnered research complied with their own theoretic principles laid out in the study. Academic researchers are using the strengths of communities, for example in recruiting research participants, but fail to meet their own standards of collaborative research in other aspects. Reviews of community participation in research outside the HIV field have generated similar results [56–59]. For example, De Las Nueces *et al*. analyzed clinical trials that used the CBPR approach and showed that only a minority reported community participation throughout the research process [57]. Research that involves community in all phases of the research still remains the exception. Based on the various options of community participation in research identified through this literature review, the authors propose a conceptual scheme of community-academic partnership in HIV research, as presented in Figure 7.

While not all HIV research needs to comply with these key principles, many studies would benefit from applying these when establishing a community-academia research partnership. Stronger involvement of community stakeholders in steps traditionally considered the area of expertise of academic researchers would ensure equal partnership, and improve the relevance, and applicability of research. In addition, researchers must assume their responsibilities beyond the dissemination of findings and support communities in the planning and implementation of interventions that result from the research. Despite shared responsibilities, all partners should remain masters in their areas of expertise in order to safeguard the added benefit of community-academia partnerships.

### Limitations of the review

One limitation of the study is the restriction of the search to publications indexed in PubMed, which could have introduced a bias. The authors recognize that additional papers, which may not have been indexed in PubMed, could have complemented the body of data. Similarly, the restriction to articles written in English might have created a bias towards countries that are using English as main language in research, thus neglecting other definitions or approaches to community involvement in research.

The results of the review reflect the information provided in the individual publications identified and analyzed. Authors might not have always explained in detail the participation of community members in individual steps of the research process due to word limitations or other restrictions, giving the impression that the community involvement was less prominent than it might have been in reality. Nonetheless, the way in which participation of community is described in the literature is indicative of the significance that researchers place on the collaboration.

## Conclusions

The results of the literature review demonstrate a diversity of approaches to community participation in HIV research, in theory and practice, and underline the need for a guiding framework for community participation in the field of HIV research. A clear definition, consistent terminology, and a framework outlining the role of community in different steps of the research process could substantially enhance and promote CPR practices. While such a framework must allow flexibility to define the specific responsibilities of each partner according to the needs and capacities, it would provide a structure to operate within. A framework of research with community participation in the HIV field would not only guide community-academia partnerships, it would also create a more structured and recognized approach which could encourage increasing funding of community-partnered research.

The authors propose a consultative process with multiple stakeholders including community and academia to identify the best approaches to HIV research with community participation moving towards commonly used terminology, criteria and processes. Such a framework will only be adopted and implemented on a broad scale if it achieves a strong consensus and is widely endorsed by relevant actors. The HIV field is characterized by strong involvement of affected communities in all areas of the HIV response. Developing a framework that provides guidance for meaningful involvement of communities in HIV research would therefore be an important step. Subsequently, the framework developed for the HIV field could be adapted and applied to other areas of research.

Besides the requirement for a framework laying out the principles and steps of community participation in research, structural changes are needed to create a facilitating environment for community-academic partnerships. Funding agencies should encourage community participation in grant application and must accommodate specific constraints of community-academia partnerships. Research grants supporting community participation in research should allow for flexibility regarding the time frame and resources, and the content of the research should take into account the diversity of communities and the changing needs within community settings. Equally, mechanisms to include communities in the ethical approval process are necessary to foster meaningful community involvement in research.

## Supplementary Material

Community-academic partnerships in HIV-related research: a systematic literature review of theory and practice
