# Supplementary material for: Community-academic partnerships in HIV-related research: a systematic literature review of theory and practice
Source: J Int AIDS Soc. 2015 Jan 27;18(1):19354. doi: 10.7448/IAS.18.1.19354 (PMC4309828; doi:10.7448/IAS.18.1.19354)
Supplement: Community-academic partnerships in HIV-related research: a systematic literature review of theory and practice [file JIAS-18-19354-s001.pdf]

Supplementary table 1 : Articles identified through PubMed search and included in the literature review

ID	Authors	Title	Year of Publication
1	Andrasik MP, Chapman CH, Clad R, Murray K, Foster J, Morris M, Parks MR, Kurth AE	Developing concurrency messages for the black community in Seattle, washington	2012
2	Apinundecha C, Laohasiriwong W, Cameron MP, Lim S	A community participation intervention to reduce HIV/AIDS stigma, Nakhon Ratchasima province, northeast Thailand	2007
3	Aronowitz T, Agbeshie E.	Nature of communication: voices of 11-14 year old African-American girls and their mothers in regard to talking about sex.	2012
4	Aronowitz T, Todd E, Agbeshie E, Rennells RE	Attitudes that affect the ability of African American preadolescent girls and their mothers to talk openly about sex	2007
5	Baptiste DR, Bhana A, Petersen I, McKay M, Voisin D, Bell C, Martinez DD	Community collaborative youth-focused HIV/AIDS prevention in South Africa and Trinidad: preliminary findings	2006
6	Berkley-Patton J, Bowe-Thompson C, Bradley-Ewing A, Hawes S, Moore E, Williams E, Martinez D, Goggin K	Taking It to the Pews: a CBPR-guided HIV awareness and screening project with black churches	2010
7	Brondani M, Moniri NR, Kerston RP	Community-Based Research among Marginalized HIV Populations: Issues of Support, Resources, and Empowerment	2012
8	Browne DC, Clubb PA, Aubrecht AM, Jackson M	Minority health risk behaviors: an introduction to research on sexually transmitted diseases, violence, pregnancy prevention and substance use	2001
9	Browne G, Browne JA, McGee F	An Ontario initiative to enhance the effectiveness of AIDS Service Organizations: Community-Linked Evaluation of AIDS Resources	2005
10	Burkhalter JE, Cahill S, Shuk E, Guidry J, Corner G, Berk A, Candelario N, Kornegay M, Lubetkin EI.	At the Intersection of HIV/AIDS and Cancer: A Qualitative Needs Assessment of Community-Based HIV/AIDS Service Organizations.	2012
11	Cashman R, Eng E, Simán F, Rhodes SD.	Exploring the sexual health priorities and needs of immigrant Latinas in the southeastern United States: a community-based participatory research approach	2011
12	Cashman SB, Adeky S, Allen AJ 3rd, Corburn J, Israel BA, Montaño J, Rafelito A, Rhodes SD, Swanston S, Wallerstein N, Eng E	The power and the promise: working with communities to analyze data, interpret findings, and get to outcomes	2008
13	Chen JC, Goetz MB, Feld JE, Taylor A, Anaya H, Burgess J, Flores Rde M, Gidwani RA, Knapp H, Ocampo EH, Asch SM	A provider participatory implementation model for HIV testing in an ED	2010
14	Chung K, Lounsbury DW	The role of power, process, and relationships in participatory research for statewide HIV/AIDS programming	2006
15	Chung PJ, Travis R Jr, Kilpatrick SD, Elliott MN, Lui C, Khandwala SB, Dancel TM, Vollandt L, Schuster MA	Acculturation and parent-adolescent communication about sex in Filipino-American families: a community-based participatory research study	2007
16	Clatts MC, Rodríguez-Díaz CE, García H, Vargas-Molina RL, Colón-López V, Pérez-Rios N, Goldsamt L, Jovet-Toledo GG	Sexually transmitted infections clinics as strategic venues for targeting high risk populations for HIV research and sexual health interventions	2011
17	Corbie-Smith G, Adimora AA, Youmans S, Muhammad M, Blumenthal C, Ellison A, Akers A, Council B, Thigpen Y, Wynn M, Lloyd SW	Project GRACE: a staged approach to development of a community-academic partnership to address HIV in rural African American communities	2010
18	Corbie-Smith G, Akers A, Blumenthal C, Council B, Wynn M, Muhammad M, Stith D	Intervention mapping as a participatory approach to developing an HIV prevention intervention in rural African American communities	2010
19	Craig Rushing S, Stephens D.	Tribal recommendations for designing culturally appropriate technology-based sexual health interventions targeting Native youth in the Pacific Northwest.	2012
20	DeCarlo P, Goldstein E	The role of community research	1999
21	Deeds BG, Castillo M, Beason Z, Cunningham SD, Ellen JM, Peralta L; Adolescent Trials Network for HIV/AIDS Interventions	An HIV prevention protocol reviewed at 15 national sites: how do ethics committees protect communities?	2008
22	Derose KP, Mendel PJ, Kanouse DE, Bluthenthal RN, Castaneda LW, Hawes-Dawson J, Mata M, Oden CW	Learning about urban congregations and HIV/AIDS: community-based foundations for developing congregational health interventions	2010
23	DiStefano AS, Hui B, Barrera-Ng A, Quitugua LF, Peters R, Dimaculangan J, Vunileva I, Tui'one V, Takahashi LM, Tanjasiri SP	Contextualization of HIV and HPV risk and prevention among Pacific Islander young adults in Southern California.	2012
24	Edwards KE, Gibson N, Martin J, Mitchell S, Andersson N.	Impact of community-based interventions on condom use in the Tłįcho region of Northwest Territories, Canada.	2011

25	Enriquez M, Cheng AL, Kelly PJ, Witt J, Coker AD, Kashubeck-West S	Development and feasibility of an HIV and IPV prevention intervention among low-income mothers receiving services in a Missouri Day Care Center	2010
26	Esau O	'Checkmating HIV&AIDS': Using chess to break the silence in the classroom	2012
27	Fauchald SK	Community-based research to explore safer sex behaviors among women: implications for CNS practice	2006
28	Fenton KA, Chinouya M, Davidson O, Copas A; MAYISHA study team	HIV testing and high risk sexual behaviour among London's migrant African communities: a participatory research study	2002
29	Ferré CD, Jones L, Norris KC, Rowley DL	The Healthy African American Families (HAAF) project: from community-based participatory research to community-partnered participatory research	2010
30	Flicker S	Who benefits from community-based participatory research? A case study of the Positive Youth Project	2008
31	Flicker S, Guta A, Larkin J, Flynn S, Fridkin A, Travers R, Pole JD, Layne C.	Survey design from the ground up: collaboratively creating the Toronto Teen Survey	2008
32	Flicker S, Skinner H, Read S, Veinot T, McClelland A, Saulnier P, Goldberg E	Falling through the cracks of the big cities: who is meeting the needs of HIV-positive youth?	2005
33	Flicker S, Wilson M, Travers R, Bereket T, McKay C, van der Meulen A, Guta A, Cleverly S, Rourke SB	Community-based research in AIDS-service organizations: what helps and what doesn't?	2009
34	Fongkaew W, Fongkaew K, Muecke M	HIV/sexual and reproductive health program for HIV prevention: the youth-adult partnership with schools approach	2006
35	Fongkaew W, Fongkaew K, Suchaxaya P	Early adolescent peer leader development in HIV prevention using youth-adult partnership with schools approach	2007
36	Fortune T, Wright E, Juzang I, Bull S	Recruitment, enrollment and retention of young black men for HIV prevention research: experiences from The 411 for Safe Text project	2010
37	Foster J, Stanek K	Cross-cultural considerations in the conduct of community-based participatory research	2007
38	Fournier B, Kipp W, Mill J, Walusimbi M	Nursing care of AIDS patients in Uganda	2007
39	Fox CE, Morford TG, Fine A, Gibbons MC	The Johns Hopkins Urban Health Institute: A collaborative response to urban health issues	2004
40	Gaudine A, Gien L, Thuan TT, Dung do V	Developing culturally sensitive interventions for Vietnamese health issues: an action research approach	2009
41	Gaudine A, Gien L, Thuan TT, Dung do V	An action research approach to developing culturally relevant interventions: the stigma of HIV in a Vietnamese community	2007
42	George C, Adam BA, Read SE, Husbands WC, Remis RS, Makoroka L, Rourke SB	The MaBwana Black men's study: community and belonging in the lives of African, Caribbean and other Black gay men in Toronto.	2012
43	Gesink D, Rink E, Montgomery-Andersen R, Mulvad G, Koch A	Developing a culturally competent and socially relevant sexual health survey with an urban Arctic community	2010
44	Gesink Law D, Rink E, Mulvad G, Koch A	Sexual health and sexually transmitted infections in the North American Arctic	2008
45	Goto K, Tiffany J, Pelto G, Pelletier D	Use of Q methodology to analyze divergent perspectives on participatory action research as a strategy for HIV/AIDS prevention among Caribbean youth	2008
46	Greene S, Tucker R, Rourke SB, Monette L, Koornstra J, Sobota M, Byers S, Hwang S, Dunn J, Guenter D, Ahluwalia A, Watson J	"Under My Umbrella": the housing experiences of HIV positive parents who live with and care for their children in Ontario	2009
47	Griffith DM, Pichon LC, Campbell B, Allen JO	YOUR Blessed Health: a faith-based CBPR approach to addressing HIV/AIDS among African Americans	2010
48	Gubrium AC, Brown EJ	Lessons learned from taking data collection to the "hood"	2006
49	Guerin PB, Allotey P, Hussein Elmi F, Baho S	Advocacy as a means to an end: assisting refugee women to take control of their reproductive health needs	2006
50	Harris GE	Practicing HIV/AIDS community-based research	2006
51	Harris GE, Larsen D	HIV peer counseling and the development of hope: perspectives from peer counselors and peer counseling recipients	2007

52	Hergenrather KC, Rhodes SD, Clark G.	Windows to work: exploring employment-seeking behaviors of persons with HIV/AIDS through Photovoice	2006
53	Hill WA, McNeely C	HIV/AIDS Disparity between African-American and Caucasian Men Who Have Sex with Men: Intervention Strategies for the Black Church	2011
54	Holgate HS, Longman C	Some peoples' psychological experiences of attending a sexual health clinic and having a sexually transmitted infection	1998
55	Hong J, Fongkaew W, Senaratana W, Tonmukayakul O	Development of a theory-based sexual and reproductive health promotion and HIV prevention program for Chinese early adolescents	2010
56	Huby G	Interpreting silence, documenting experience: an anthropological approach to the study of health service users' experience with HIV/AIDS care in Lothian, Scotland	1997
57	Jenkin P, Koch T, Kralik D.	The experience of fatigue for adults living with HIV	2006
58	Jirapaet V	Effects of an empowerment program on coping, quality of life, and the maternal role adaptation of Thai HIV-infected mothers	2000
59	Johnson CE, Ali SA, Shipp MP	Building community-based participatory research partnerships with a Somali refugee community	2009
60	Kelly J, Luxford Y	Yaitya tirka madlanna warratinna: exploring what sexual health nurses need to know and do in order to meet the sexual health needs of young Aboriginal women in Adelaide	2007
61	Kesby M	Participatory diagramming as a means to improve communication about sex in rural Zimbabwe: a pilot study	2000
62	Kielhofner G, Braveman B, Finlayson M, Paul-Ward A, Goldbaum L, Goldstein K	Outcomes of a vocational program for persons with AIDS	2004
63	Kobetz E, Menard J, Hazan G, Koru-Sengul T, Joseph T, Nissan J, Barton B, Blanco J, Kornfeld J	Perceptions of HPV and cervical cancer among Haitian immigrant women: implications for vaccine acceptability.	2011
64	Kovacs PJ	Participatory action research and hospice: a good fit	2000
65	Kubicek K, Beyer WH, McNeeley M, Weiss G, Ultra Omni LF, Kipke MD	Community-Engaged Research to Identify House Parent Perspectives on Support and Risk Within the House and Ball Scene.	2011
66	Lee H, Pollock G, Lubek I, Niemi S, O'Brien K, Green M, Bashir S, Braun E, Kros S, Huot V, Ma V, Griffiths N, Dickson B, Pring N, Sohkurt Huon-Ribeil K, Lim N, Turner J, Winkler C, Wong ML, Van Merode T, Dy BC, Prem S, Idema R	Creating new career pathways to reduce poverty, illiteracy and health risks, while transforming and empowering Cambodian women's lives	2010
67	Lightfoot AF, Woods BA, Jackson M, Riggins L, Krieger K, Brodie K, Gray P, Howard DL	"In My House": Laying the Foundation for Youth HIV Prevention in the Black Church.	2012
68	Lindsey E, Stajduhar K	From rhetoric to action: establishing community participation in AIDS-related research	1998
69	Lindsey E, Stajduhar K, McGuinness L	Examining the process of community development	2001
70	Lloyd SW, Ferguson YO, Corbie-Smith G, Ellison A, Blumenthal C, Council BJ, Youmans S, Muhammad MR, Wynn M, Adimora A, Akers A	The role of public schools in HIV prevention: perspectives from African Americans in the rural South.	2012
71	Logie C, James L, Tharao W, Loutfy MR	Opportunities, ethical challenges, and lessons learned from working with peer research assistants in a multi-method HIV community-based research study in Ontario, Canada	2012
72	Loue S, Lloyd L, Loh L.	HIV prevention in U.S. Asian Pacific Islander communities: an innovative approach	1996
73	Marcus MT, Walker T, Swint JM, Smith BP, Brown C, Busen N, Edwards T, Liehr P, Taylor WC, Williams D, von Sternberg K	Community-based participatory research to prevent substance abuse and HIV/AIDS in African-American adolescents	2004
74	Martin RE, Murphy K, Chan R, Ramsden VR, Granger-Brown A, Macaulay AC, Kahlon R, Ogilvie G, Hislop TG	Primary health care: applying the principles within a community-based participatory health research project that began in a Canadian women's prison	2009
75	Maticka-Tyndale E	Bridging theory and practice in HIV prevention for rural youth, Nigeria.	2012
76	McQuiston C, Parrado EA, Martínez AP, Uribe L	Community-based participatory research with Latino community members: horizonte Latino	2005
77	Mensah MN, Waugh T, Lavoie R, Dumas J, Bernier M, Garneau MJ, Giroux C, Otis J	"The VIHsibilite Project": HIV-positive people in the Quebec press and community responses	2008
78	Mill J, Edwards N, Jackson R, Austin W, MacLean L, Reintjes F	Accessing health services while living with HIV: intersections of stigma	2009

79	Mill JE	Shrouded in secrecy: breaking the news of HIV infection to Ghanaian women	2003
80	Mill JE	I'm not a "basabasa" woman: an explanatory model of HIV illness in Ghanaian women	2001
81	Mill JE, Edwards N, Jackson RC, MacLean L, Chaw-Kant J	Stigmatization as a social control mechanism for persons living with HIV and AIDS	2010
82	Mill JE, Ogilvie LD	Establishing methodological rigour in international qualitative nursing research: a case study from Ghana	2003
83	Mooney-Somers J, Erick W, Scott R, Akee A, Kaldor J, Maher L	Enhancing Aboriginal and Torres Strait Islander young people's resilience to blood-borne and sexually transmitted infections: findings from a community-based participatory research project	2009
84	Mooney-Somers J, Maher L.	The Indigenous Resiliency Project: a worked example of community-based participatory research	2009
85	Mooney-Somers J, Olsen A, Erick W, Scott R, Akee A, Kaldor J, Maher L	Learning from the past: young Indigenous people's accounts of blood-borne viral and sexually transmitted infections as resilience narratives	2011
86	Morisky DE, Ang A, Coly A, Tiglao TV	A model HIV/AIDS risk reduction programme in the Philippines: a comprehensive community-based approach through participatory action research	2004
87	Morisky DE, Malow RM, Tiglao TV, Lyu SY, Vissman AT, Rhodes SD	Reducing sexual risk among Filipina female bar workers: effects of a CBPR-developed structural and network intervention	2010
88	Mosavel M, Simon C, van Stade D, Buchbinder M	Community-based participatory research (CBPR) in South Africa: engaging multiple constituents to shape the research question	2005
89	Nokes KM, Hughes V, Santos R, Bang H.	Creating a Paper-Based Personal Health Record for HIV-Infected Persons.	2012
90	Nyamathi A, Koniak-Griffin D, Tallen L, González-Figueroa E, Levson L, Mosley Y, Dominick E, Anderson NL	Use of community-based participatory research in preparing low income and homeless minority populations for future HIV vaccines	2004
91	Nyamathi AM, Sinha S, Ganguly KK, William RR, Heravian A, Ramakrishnan P, Greengold B, Ekstrand M, Rao PV	Challenges experienced by rural women in India living with AIDS and implications for the delivery of HIV/AIDS care	2011
92	Nyamathi AM, William RR, Ganguly KK, Sinha S, Heravian A, Albarrán CR, Thomas A, Greengold B, Ekstrand M, Ramakrishna P, Rao PR	Perceptions of Women Living with AIDS in Rural India Related to the Engagement of HIV-Trained Accredited Social Health Activists for Care and Support	2010
93	Ogden R	Ethical review in community-based HIV/AIDS research	1999
94	Operario D, Nemoto T, Ng T, Syed J, Mazarei M	Conducting HIV interventions for Asian Pacific Islander men who have sex with men: challenges and compromises in community collaborative research	2005
95	Pinto RM	Community perspectives on factors that influence collaboration in public health research	2009
96	Pinto RM, da Silva SB, Penido C, Spector AY	International participatory research framework: triangulating procedures to build health research capacity in Brazil	2011
97	Pinto RM, Melendez RM, Spector AY	Male-to-Female Transgender Individuals Building Social Support and Capital From Within a Gender-Focused Network	2008
98	Poindexter CC, Lane TS	Choices and voices: participation of people with HIV on Ryan White Title II Consumer Advisory Boards	2003
99	Power R, Dale A, Jones S	Towards a process evaluation model for community-based initiatives aimed at preventing the spread of HIV amongst injecting drug users	1991
100	Pratt RJ, Pellowe CM, Juvekar SK, Potdar NS, Weston AJ, Joykutty A, Robinson N, Loveday HP	Kaleidoscope: a 5-year action research project to develop nursing confidence in caring for patients with HIV disease in west India	2001
101	Radda KE, Schensul JJ, Disch WB, Levy JA, Reyes CY	Assessing human immunodeficiency virus (HIV) risk among older urban adults: a model for community-based research partnership	2003
102	Ramjee G, Coumi N, Dladla-Qwabe N, Ganesh S, Gappoo S, Govinden R, Guddera V, Maharaj R, Moodley J, Morar N, Naidoo S, Palanee T	Experiences in conducting multiple community-based HIV prevention trials among women in KwaZulu-Natal, South Africa	2010
103	Remple VP, Johnston C, Patrick DM, Tyndall MW, Jolly AM	Conducting HIV/AIDS research with indoor commercial sex workers: reaching a hidden population	2007
104	Rhodes SD, Eng E, Hergenrather KC, Remnitz IM, Arceo R, Montaño J, Alegría-Ortega J	Exploring Latino men's HIV risk using community-based participatory research	2007
105	Rhodes SD, Hergenrather KC, Aronson RE, Bloom FR, Felizzola J, Wolfson M, Vissman AT, Alonzo J, Boeving Allen A, Montano J, McGuire J	Latino men who have sex with men and HIV in the rural south-eastern USA: findings from ethnographic in-depth interviews	2010

106	Rhodes SD, Hergenrather KC, Duncan J, Ramsey B, Yee LJ, Wilkin AM	Using community-based participatory research to develop a chat room-based HIV prevention intervention for gay men.	2007
107	Rhodes SD, Hergenrather KC, Duncan J, Vissman AT, Miller C, Wilkin AM, Stowers J, Eng E	A pilot intervention utilizing Internet chat rooms to prevent HIV risk behaviors among men who have sex with men	2010
108	Rhodes SD, Hergenrather KC, Montaño J, Remnitz IM, Arceo R, Bloom FR, Leichliter JS, Bowden WP	Using community-based participatory research to develop an intervention to reduce HIV and STD infections among Latino men	2006
109	Rhodes SD, Hergenrather KC, Vissman AT, Stowers J, Davis AB, Hannah A, Alonzo J, Marsiglia FF	Boys must be men, and men must have sex with women: a qualitative CBPR study to explore sexual risk among African American, Latino, and White gay men and MSM	2011
110	Rhodes SD, Hergenrather KC, Wilkin A, Alegría-Ortega J, Montaño J	Preventing HIV infection among young immigrant Latino men: results from focus groups using community-based participatory research	2006
111	Rhodes SD, Hergenrather KC, Yee LJ, Wilkin AM, Clarke TL, Wooldredge R, Brown M, Davis AB	Condom acquisition and preferences within a sample of sexually active gay and bisexual men in the southern United States	2007
112	Rhodes SD, Kelley C, Simán F, Cashman R, Alonzo J, McGuire J, Wellendorf T, Hinshaw K, Allen AB, Downs M, Brown M, Martínez O, Duck S, Reboussin B.	Using community-based participatory research (CBPR) to develop a community level HIV prevention intervention for Latinas: a local response to a global challenge.	2012
113	Rhodes SD, Malow RM, Jolly C	Community-based participatory research: a new and not-so-new approach to HIV/AIDS prevention, care, and treatment	2010
114	Rhodes SD, McCoy TP, Vissman AT, DiClemente RJ, Duck S, Hergenrather KC, Foley KL, Alonzo J, Bloom FR, Eng E	A randomized controlled trial of a culturally congruent intervention to increase condom use and HIV testing among heterosexually active immigrant Latino men	2011
115	Rhodes SD, Tanner A, Duck S, Aronson RE, Alonzo J, Garcia M, Wilkin AM, Cashman R, Vissman AT, Miller C, Kroeger K, Naughton MJ	Female sex work within the rural immigrant Latino community in the Southeast United States: an exploratory qualitative community-based participatory research study	2012
116	Rhodes SD, Vissman AT, Stowers J, Miller C, McCoy TP, Hergenrather KC, Wilkin AM, Reece M, Bachmann LH, Ore A, Ross MW, Hendrix E, Eng E	A CBPR partnership increases HIV testing among men who have sex with men (MSM): outcome findings from a pilot test of the CyBER/testing internet intervention	2011
117	Rhodes SD, Yee LJ, Hergenrather KC	A community-based rapid assessment of HIV behavioural risk disparities within a large sample of gay men in southeastern USA: a comparison of African American, Latino and white men	2006
118	Richards J, Mousseau A.	Community-based participatory research to improve preconception health among Northern Plains American Indian adolescent women.	2012
119	Rink E, FourStar K, Medicine Elk J, Dick R, Jewett L, Gesink D.	Pregnancy prevention among American Indian men ages 18 to 24: the role of mental health and intention to use birth control.	2012
120	Rink E, Gesink Law D, Montgomery-Andersen R, Mulvad G, Koch A	The practical application of community-based participatory research in Greenland: initial experiences of the Greenland Sexual Health Study	2009
121	Rios-Ellis B, Espinoza L, Bird M, Garcia M, D'Anna LH, Bellamy L, Scolari R.	Increasing HIV-related knowledge, communication, and testing intentions among Latinos: Protege tu Familia: Hazte la Prueba	2010
122	Robinson BB, Uhl G, Miner M, Bockting WO, Scheltema KE, Rosser BR, Westover B	Evaluation of a sexual health approach to prevent HIV among low income, urban, primarily African American women: results of a randomized controlled trial	2002
123	Rosenthal WA, Khalil DD	Exploring the challenges of implementing Participatory Action Research in the context of HIV and poverty	2010
124	Roy CM, Cain R	The involvement of people living with HIV/AIDS in community-based organizations: contributions and constraints	2001
125	Rudolph AE, Standish K, Amesty S, Crawford ND, Stern RJ, Badillo WE, Boyer A, Brown D, Ranger N, Orduna JM, Lasenburg L, Lippek S, Fuller CM	A community-based approach to linking injection drug users with needed services through pharmacies: an evaluation of a pilot intervention in New York City	2010
126	Sanstad KH, Stall R, Goldstein E, Everett W, Brousseau R	Collaborative community research consortium: a model for HIV prevention	1999
127	Satinsky S, Fisher C, Stupiansky N, Dodge B, Alexander A, Herbenick D, Reece M	Sexual compulsivity among men in a decentralized MSM community of the Midwestern United States	2008
128	Schensul SL, Nastasi BK, Verma RK	Community-based research in India: a case example of international and transdisciplinary collaboration	2006
129	Schnarrs PW, Rosenberger JG, Satinsky S, Brinegar E, Stowers J, Dodge B, Reece M	Sexual compulsivity, the Internet, and sexual behaviors among men in a rural area of the United States	2010
130	Schoepf BG	AIDS action-research with women in Kinshasa, Zaire	1993
131	Schoepf BG	AIDS, sex and condoms: African healers and the reinvention of tradition in Zaire	1992
132	Seeley JA, Kengeya-Kayondo JF, Mulder DW	Community-based HIV/AIDS research--whither community participation? Unsolved problems in a research programme in rural Uganda	1992

133	Shannon K, Bright V, Allinott S, Alexson D, Gibson K, Tyndall MW; Maka Project Partnership	Community-based HIV prevention research among substance-using women in survival sex work: the Maka Project Partnership	2007
134	Shannon K, Kerr T, Allinott S, Chettiar J, Shoveller J, Tyndall MW	Social and structural violence and power relations in mitigating HIV risk of drug-using women in survival sex work	2007
135	Spirig R, Nicca D, Voggensperger J, Unger M, Werder V, Niepmann S	The Advanced Nursing Practice Team as a model for HIV/AIDS caregiving in Switzerland	2004
136	Stajduhar KI, Lindsey E	Home away from home: essential elements in developing housing options for people living with HIV/AIDS	1999
137	Stajduhar KI, Lindsey E, McGuinness L	A qualitative evaluation of an HIV/AIDS respite care service in Victoria, Canada	2002
138	Stevens PE, Hall JM	Participatory action research for sustaining individual and community change: a model of HIV prevention education	1998
139	Taylor RR, Braveman B, Hammel J	Developing and evaluating community-based services through participatory action research: two case examples	2004
140	Teti M, Murray C, Johnson L, Binson D	Photovoice as a community-based participatory research method among women living with HIV/AIDS: ethical opportunities and challenges	2012
141	Travers R, Wilson M, McKay C, O'Campo P, Meagher A, Hwang SW, Parris UJ, Cowan L	Increasing accessibility for community participants at academic conference	2008
142	Travers R, Wilson MG, Flicker S, Guta A, Bereket T, McKay C, van der Meulen A, Cleverly S, Dickie M, Globerman J, Rourke SB	The Greater Involvement of People Living with AIDS principle: theory versus practice in Ontario's HIV/AIDS community-based research sector	2008
143	Twinn S	Developments in nursing practice in primary health care in Hong Kong: opportunities and challenges	2001
144	Ubaidullah M	Social vaccine for HIV prevention: a study on truck drivers in South India	2005
145	Visser M, Finestone M, Sikkema K, Boeving-Allen A, Ferreira R, Eloff I, Forsyth B.	Development and piloting of a mother and child intervention to promote resilience in young children of HIV-infected mothers in South Africa.	2012
146	Visser M, Mundell J, de Villiers A, Sikkema K, Jeffery B	Development of structured support groups for HIV-positive women in South Africa	2005
147	Visser MJ	Life skills training as HIV/AIDS preventive strategy in secondary schools: evaluation of a large-scale implementation process	2005
148	Vissman AT, Eng E, Aronson RE, Bloom FR, Leichliter JS, Montaño J, Rhodes SD	What do men who serve as lay health advisers really do?: Immigrant Latino men share their experiences as Navegantes to prevent HIV	2009
149	Waterman H, Griffiths J, Gellard L, O'Keefe C, Olang G, Ayuyo J, Obwanda E, Ogwethe V, Ondiege J	Power brokering, empowering, and educating: the role of home-based care professionals in the reduction of HIV-related stigma in Kenya.	2007
150	Weeks MR, Liao S, Li F, Li J, Dunn J, He B, He Q, Feng W, Wang Y	Challenges, strategies, and lessons learned from a participatory community intervention study to promote female condoms among rural sex workers in Southern China	2010
151	Williams JK, Wyatt GE, Wingood G	The four Cs of HIV prevention with African Americans: crisis, condoms, culture, and community	2010
152	Williams MV, Palar K, Derose KP	Congregation-based programs to address HIV/AIDS: elements of successful implementation	2011
153	Willms DG, Arratia MI, Makondesa P	Can interfaith research partnerships develop new paradigms for condom use and HIV prevention? The implementation of conceptual events in Malawi results in a 'spiritualised condom'	2011
154	Wingood GM, Simpson-Robinson L, Braxton ND, Raiford JL	Design of a faith-based HIV intervention: successful collaboration between a university and a church.	2011
155	Wong FY, Crisostomo VA, Bao D, Smith BD, Young D, Huang ZJ, Buchholz ME, Frangos SN; MATH Study Consortium	Development and implementation of a collaborative, multistakeholder research and practice model on HIV prevention targeting Asian/Pacific Islander men in the United States who have sex with men	2010
156	Wood L	Every teacher is a researcher!': Creating indigenous epistemologies and practices for HIV prevention through values-based action research.	2012
157	Worthington C, Este D, Strain KL, Huffey N.	African immigrant views of HIV service needs: Gendered perspectives.	2012
158	Worthington C, Jackson R, Mill J, Prentice T, Myers T, Sommerfeldt S	HIV testing experiences of Aboriginal youth in Canada: service implications.	2010
159	Yancey EM, Mayberry R, Armstrong-Mensah E, Collins D, Goodin L, Cureton S, Trammell EH, Yuan K	The community-based participatory intervention effect of "HIV-RAAP".	2012
